# Mitochondrial DNA Regionalism and Historical Demography in the Extant Populations of *Chirocephalus kerkyrensis* (Branchiopoda: Anostraca)

**DOI:** 10.1371/journal.pone.0030082

**Published:** 2012-02-17

**Authors:** Valerio Ketmaier, Federico Marrone, Giuseppe Alfonso, Kirsten Paulus, Annika Wiemann, Ralph Tiedemann, Graziella Mura

**Affiliations:** 1 Unit of Evolutionary Biology/Systematic Zoology, Institute of Biochemistry and Biology, University of Potsdam, Potsdam, Germany; 2 Department of Biology and Biotechnology “Charles Darwin”, University of Rome “Sapienza”, Rome, Italy; 3 Department of Environmental Biology and Biodiversity, University of Palermo, Palermo, Italy; 4 Laboratory of Zoogeography and Fauna, Di. S. Te. B.A., University of Salento, Lecce, Italy; 5 Department of Biology, Institute of Forensic Science, State Office of Criminal Investigation, Hannover, Germany; Ecole Normale Supérieure de Lyon, France

## Abstract

**Background:**

Mediterranean temporary water bodies are important reservoirs of biodiversity and host a unique assemblage of diapausing aquatic invertebrates. These environments are currently vanishing because of increasing human pressure. *Chirocephalus kerkyrensis* is a fairy shrimp typical of temporary water bodies in Mediterranean plain forests and has undergone a substantial decline in number of populations in recent years due to habitat loss. We assessed patterns of genetic connectivity and phylogeographic history in the seven extant populations of the species from Albania, Corfu Is. (Greece), Southern and Central Italy.

**Methodology/Principal Findings:**

We analyzed sequence variation at two mitochondrial DNA genes (Cytochrome Oxidase I and 16s rRNA) in all the known populations of *C. kerkyrensis*. We used multiple phylogenetic, phylogeographic and coalescence-based approaches to assess connectivity and historical demography across the whole distribution range of the species. *C. kerkyrensis* is genetically subdivided into three main mitochondrial lineages; two of them are geographically localized (Corfu Is. and Central Italy) and one encompasses a wide geographic area (Albania and Southern Italy). Most of the detected genetic variation (≈81%) is apportioned among the aforementioned lineages.

**Conclusions/Significance:**

Multiple analyses of mismatch distributions consistently supported both past demographic and spatial expansions with the former predating the latter; demographic expansions were consistently placed during interglacial warm phases of the Pleistocene while spatial expansions were restricted to cold periods. Coalescence methods revealed a scenario of past isolation with low levels of gene flow in line with what is already known for other co-distributed fairy shrimps and suggest drift as the prevailing force in promoting local divergence. We recommend that these evolutionary trajectories should be taken in proper consideration in any effort aimed at protecting Mediterranean temporary water bodies.

## Introduction

With 49 species, *Chirocephalus* is the most speciose anostracan genus in the Palaearctics, and the second one worldwide [1], [2]. Based on the morphology of antennae and penes five species-groups have been identified within the genus (bairdi, diaphanus, spinicaudatus, pristicephalus and sinensis) [3], [4], [5]. Recent molecular evidences re-instated *Pristicephalus* as an independent genus [6], thus bringing the species groups into which the genus *Chirocephalus* is divided to four; three of these (bairdi, diaphanus and spinicaudatus) are distributed in the West-Palaearctic area. The Ponto-Mediterranean bairdi-group is distributed along the northern coasts of the east-Mediterranean basin, from peninsular Italy to Jordan and Israel, through the Balkan Peninsula and Asia Minor [5], and includes eight species and one subspecies [3], [7], [8], [9].

*Chirocephalus kerkyrensis* Pesta, 1936 is the westernmost representative of the bairdi-species group. The species, first identified in the temporary water bodies of Corfu Island (Greece) [10], [11], was thought to be confined to that island until the second half of the XX century, when it was recorded in Central Italy [12]. In that area the species appeared to be limited to a few localities south of Rome [13]–[18]. In spite of extensive sampling efforts, *C. kerkyrensis* has never been recorded north of Rome, where it seems to be replaced by the widespread and more euryoecious congener *C. diaphanus* Prévost, 1803 [15], [19]–[22]. During recent samplings the species has been found for the first time in Southern Italy (Basilicata) [23] and in the Balkan area (Albania; Alfonso & Belmonte unpublished data). The species typically inhabits temporary water bodies in oak or mixed woods at the sea level, the so-called Mediterranean plain forest, or pools in meadows bordering it. The Albanian and the Southern Italian sites, however, are located in a karstic area (the former) and at a moderate altitude between about 100 and 500 m above the sea level (both).

**Figure 1:**
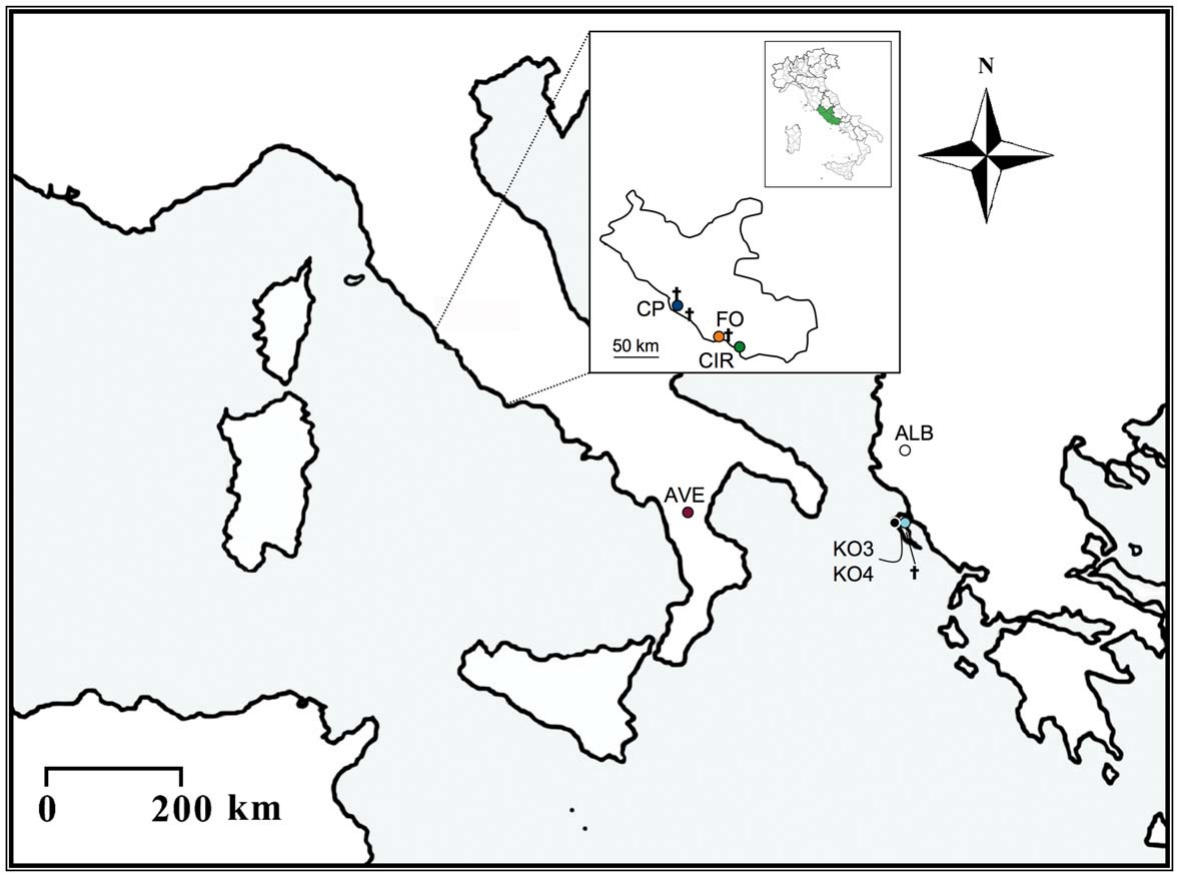
Geographic origin of the *C. kerkyrensis* populations included in the study. Population codes match those in Table 1; colors identify the different sampling locations. Crosses indicate locations where the species was known to occur but had nowadays gone extinct because of human activities. The part of Central Italy corresponding to the Latium region is zoomed in and shown in the inlet.

Temporary water bodies are extremely important reservoirs of biodiversity in the circum – Mediterranean area due to the instability of the permanent hydrographical surface network. A directive from the European Union (Council Directive 92/43/EEC) recognized these habitats as worthy of special conservation efforts; this view was further ratified by the Ramsar Convention on Wetlands (Resolution VIII.33/2002) [24]. In spite of their naturalistic value, temporary aquatic systems are currently heavily impacted by human activities. This applies particularly to those ponds located in lowlands, where the human pressure is extremely intense [25]. Due to expanding urbanization and land use, most of the original habitat of *C. kerkyrensis* has vanished and consequently many of the populations formerly known have progressively disappeared [25]. The species is nowadays considered rare being left with just seven known populations (three in Central Italy, one in Southern Italy, one in Albania and two on Corfu Is.; Figure 1).

Here we examine the geographical distribution of mitochondrial lineages in *C. kerkyrensis* across its entire distribution range by including all its extant populations. We sequenced fragments of two mitochondrial DNA (mtDNA) genes - Cytochrome Oxidase I (COI) and 16s rRNA (16S) - which have proved useful in addressing questions at the population level in anostracans [26], [27], [28]. Large branchiopods inhabiting temporary freshwater bodies represent an evolutionary paradox as they are generally characterized by high levels of regional subdivision, in spite of the theoretical potential for long distance dispersal of the resting eggs (cysts) they produce. As a matter of fact, these cysts can withstand prolonged droughts and are easily transported passively by e.g. waterbirds [29], [30]. On the other hand, it shouldn't be overlooked that having the potential to be passively transported over long distances does not necessarily imply that such a phenomenon would consistently happen and translate into substantial gene flow among populations. First, relying on secondary vectors for dispersal might be a limiting factor *per se*. Second, the essentially scattered distribution of Mediterranean temporary water bodies (and hence of the species inhabiting them) further contributes to hamper gene flow. Even when cysts of a given species are transported from pond to pond, donor and recipient water bodies must be inhabited by the same species for effective exchanges of genes across locations to happen.

We wanted to determine the degree of genetic structuring in *C. kerkyrensis* to test whether the aforementioned paradox is valid or not within a species with a relatively wide, yet fragmented, geographical distribution. We show how our molecular data are useful to clarify some taxonomic vagaries and to infer levels and patterns of genetic connectivity across the whole *C. kerkyrensis* geographic range. By revealing the degree of genetic uniqueness within the species and how this is distributed geographically we aim to further contribute to the understanding of the evolutionary trajectories in Mediterranean diapausing invertebrates. Such knowledge is an essential prerequisite for the set-up of an adequate management of the fragile and imperiled habitat represented by temporary ponds of plain and low altitude Mediterranean forests.

## Results

### Sequence variation

We sequenced about 1 kb of mtDNA for each of the 93 individuals included in the study; the final alignment includes 560 bp for the COI gene and 465 bp for the 16S gene for a total length of 1025 bp. We observed only a few gaps in the final alignment, all confined to the 16S partition and to ingroup vs. outgroups comparisons. Levels of sequence variability are higher in COI than in 16S (24.8% vs. 18.5%) but percentages of parsimony informative sites are comparable across the two genes (39.5% vs. 32.5%). COI 3^rd^ codon position is by far the most variable partition with 62.5% of variable sites (23.5% also parsimony informative). We found only a single amino acid substitution at the ingroup level in the COI dataset. Sequences are generally A+T rich (63.9%) and anti-G biased (19%); this pattern is particularly evident in COI 3^rd^ codon positions (A+T = 87.1%; G = 4.7%). Multiple *χ* ^2^ tests of homogeneity of base frequencies failed to detect any significant differences whatever data partition considered (both genes together and separately and for COI each codon position separately). Estimates of mtDNA diversity are reported in Table 1. Haplotype diversity (*h*) ranges from 0.200 (CP) to 0.777 (KO3); mean number of pairwise differences between all pairs of haplotypes (*π*) varies from 0.668 (AVE) to 7.688 (KO3).

**Table 1:** Sampling localities, designations and mtDNA diversity estimates (N: sample size; H: n of haplotypes; *h*: haplotype diversity; *π*: mean number of pairwise differences between all pairs of haplotypes; *π*_n_: nucleotide diversity) for the seven populations of *C. kerkyrensis* included in the study.

		Geographical coordinates						
Locality	Code	Latitude N	Longitude E	N	H	*h*	*π*	*π_n_* (%)
Cepa, Dumre, Albania – Merhojes Pond	ALB	40.944°	19.866°	16	5	0.650	1.975	0.19
Kokkini, Corfu Is., Greece – Pond KO3	KO3	39.616°	19.820°	10	6	0.777	7.688	0.75
Kokkini, Corfu Is., Greece – Pond KO4	KO4	39.628°	19.831°	14	4	0.626	0.868	0.08
Francavilla sul Sinni, Basilicata, Southern Italy – Pond Avena	AVE	40.050°	16.245°	20	3	0.352	0.668	0.06
Circeo National Park, Latina, Central Italy- Pond T3	CIR	41.348°	13.047°	13	5	0.730	5.256	051
Nettuno, Rome, Central Italy – Bosco di Foglino	FO	41.441°	12.732°	10	4	0.644	2.466	0.24
Castel Porziano, Rome, Central Italy – Pond A1	CP	41.705°	12.396°	10	2	0.200	1.000	0.09

### Phylogeography

**Figure 2:**
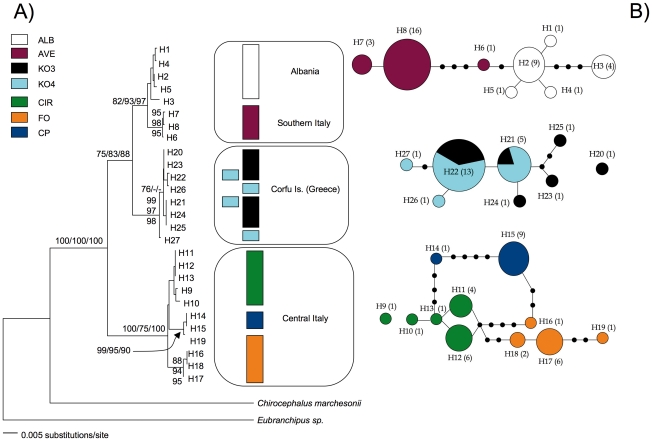
Phylogenetic and phylogeographic relationships in *C. kerkyrensis*. Colors are as in Figure 1; haplotype codes match those in Table 2. In A) the ML haplotype phylogram is shown. Numbers at nodes are statistical supports for the Bayesian, NJ and ML searches (first, second and third value, respectively); only values ≥75% are reported. The three geographical clusters are described in the text. Panel B) depicts the haplotype networks for the three geographical clusters. Only connections with a probability ≥95% are shown. The relative size of circles is proportional to the number of individuals (indicated by the numbers in parentheses) carrying that particular haplotype; black dots represent missing haplotypes. Haplotypes are always one mutational step away from each other irrespective of the length of the branch between them.

The 93 individual of *C. kerkyrensis* sequenced for the study revealed a total of 27 unique haplotypes (both genes combined). Table 2 shows their frequency in the seven study populations. Number of haplotypes per population ranges from two (CP) to six (KO3); most of the scored haplotypes are unique to single populations with the only exception of H21 and H22 that are shared between KO3 and KO4. Phylogenetic and phylogeographic analyses produced largely congruent results (Figure 2). Phylogenetic searches were based on the 27 unique haplotypes and on the GTR+I+Γ model selected by MODELTEST (proportion of invariable sites = 0.615; shape parameter α = 0.912). Three main haplogroups (Central Italy, Southern Italy/Albania, Corfu Is.) were consistently retrieved with good statistical support in the ML, NJ and Bayesian searches. A Central Italian haplogroup is placed basal to Greek and Albanian/Southern Italian haplotypes (Figure 2A). The statistical parsimony procedure yielded a network whit the same haplogroups as in the phylogenetic tree (Figure 2B); these could not be linked to one another based on the 95% parsimony criterion. Similarly, haplotypes H9 (CIR) and H20 (KO3) could not be connected to any of the identified haplogroups because differed from them by a number of substitutions (14–28) exceeding the maximum number of connection steps (13) allowed by the 95% parsimony criterion in TCS.

**Table 2:** Haplotype frequency in the populations included in the study.

Hapl./Pop.	ALB	KO3	KO4	AVE	CIR	FO	CP	Tot.
H1	1							1
H2	9							9
H3	4							4
H4	1							1
H5	1							1
H6				1				1
H7				3				3
H8				16				16
H9					1			1
H10					1			1
H11					4			4
H12					6			6
H13					1			1
H14							1	1
H15							9	9
H16						1		1
H17						6		6
H18						2		2
H19						1		1
H20		1						1
H21		1	4					5
H22		5	8					13
H23		1						1
H24		1						1
H25		1						1
H26			1					1
H27			1					1
Tot.	16	10	14	20	13	10	10	93

For population codes see Table 1 and Figure 1.

### Population structure and historical demography

Pairwise *F*_ST_ values (Table 3) range between -0.020 (KO3 vs. KO4) and 0.703 (CP vs. AVE); all but the KO3 vs. KO4 comparison are larger than 0.240 and significant after sequential Bonferroni correction. Table 4 reports the AMOVA results. Genetic variance was found to be very high and significant (*F*_ST_ = 0.914; *P* = 0.000) when populations were analyzed without any *a priori* grouping (Table 4A). Based on the pairwise *F*_ST_ values and phylogeographic results, we then ran a grouped AMOVA with populations sorted in three main groups (Albania+Southern Italy; Corfu Is.; Central Italy). AMOVA recovered significant population structure at each hierarchical level with the vast majority of the detected variation being apportioned among groups (80.59%; *F*_CT_ = 0.806; *P* = 0.006).

**Table 3:** Pairwise *F*_ST_ values among populations; all values but the KO3 vs. KO4 comparison are significant after sequential Bonferroni correction (initial α = 0.0023).

Pop.	AVE	ALB	KO3	KO4	CIR	FO	CP
AVE	-						
ALB	0.508	-					
KO3	0.476	0.292	-				
KO4	0.525	0.361	−0.020	-			
CIR	0.481	0.311	0.246	0.322	-		
FO	0.532	0.352	0.288	0.365	0.309	-	
CP	0.703	0.540	0.511	0.562	0.512	0.577	-

**Table 4:** Hierarchical analysis of molecular variance (AMOVA); in A) populations were treated as belonging to a single gene pool, while in B) populations were assigned to three groups (Albania+Southern Italy, Corfu Is., Central Italy) based on phylogenetic and phylogeographic analyses (see text).

Source of variation	Sum of squares (d.f.)	Variance component	P	Fixation index	% variation
A)					
Among populations	1071.6 (6)	Va = 13.5	0.000	*F*_ST_ = 0.914	91.45
Within populations	108.5 (86)	Vb = 1.26	-	-	-
B)					
Among groups	952.5 (2)	Va = 14.52	0.006	*F*_CT_ = 0.806	80.59
Among populations	119.6 (4)	Vb = 2.23	0.000	*F*_SC_ = 0.639	12.40
Within populations	108.5 (86)	Vc = 1.26	0.000	*F*_ST_ = 0.929	7.01

The multiple spatial autocorrelation analyses revealed significant positive spatial genetic structure for each group/distance class tested. Central Italian populations (distance class size = 10 km) showed significant positive values (*r* = 0.362, *P* = 0.001) up to 23 km (point of *x*-intercept). The point of *x*-intercept is 81 km for Central Italian+Greek populations with positive and significant values before it (*r* = 0.162, *P* = 0.001; distance class = 50 km). Albanian+Southern Italian populations and all populations together (distance class size = 100 km in both circumstances) showed significant positive values before 307 and 175 km, respectively (*r* = 0.396 and 0.256, *P* always = 0.001). Finally, the Mantel test revealed no sign of a significant IBD among populations (*R* = 0.118; *P* = 0.163).

**Figure 3:**
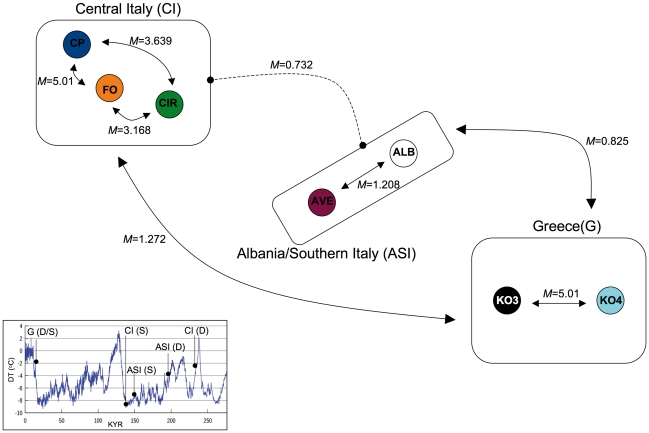
Historical demography in *C. kerkyrensis*. Schematic representation of gene flow (*M*) between and within haplogroups (arrows) as inferred by the coalescence-based method in MDIV. Gene flow estimate is reported also for the Central Italian vs. Albanian/Southern Italian comparison (dashed line) even though a sister relationship between the two haplogroups was not supported in any phylogenetic or phylogeographic analysis. The bottom left insert shows temperature variation (ΔT°C) over the last 250 Kyr (redrawn from [78]) with superimposed the age of demographic (D) and spatial (S) expansions for the Central Italian (CI), Greek (G) and Albanian/Southern Italian (ASI) haplogroups as inferred by the mismatch analyses detailed in Table 5.

Summary statistics of mismatch parameters for both demographic and spatial expansions are shown in Table 5. The observed mean number of differences at the population level varies between 0.668 (AVE) and 7.689 (KO3); at the group level the observed mean number of differences are similar within Albanian+Southern Italian and Greek lineages but higher within the Central Italian ones. When all populations are considered simultaneously the value reaches 25.656. Mismatch distributions for most of the populations/groups of populations do not differ from the purely demographic model with the only exceptions of KO3, CP and all populations together (0.04≥*P_SSD_*≥0.001); no deviations from the expected mismatch distributions are observed when the same populations/groups of populations were tested for the spatial expansion model (0.578≥*P_SSD_*≥0.061). The analyses of mismatch distributions were thus more effective in supporting spatial over purely demographic expansions. Considering the demographic model, expansions were always preceded by severe reductions in population sizes and resulted in high female effective population sizes (2.5×10^5^≥*N_1_*≥12,750). Estimates of times since expansion yielded roughly congruent figures for the two models with the exceptions of AVE (8,350–75,000 years), KO3 and CP (but for these two populations the demographic expansion model was rejected). When populations were pooled into three groups Albanian+Southern Italian and Central Italian populations had similar ages (192,375–230,650; 141,400–150,150 years for the demographic and spatial models respectively) and had greater ages than the Greek populations (≈14,400 years for both models; Figure 3). Age estimates of all populations together are largely congruent between the two models (1.07–1.15×10^5^ years). Under the spatial expansion model, spreading of populations relied on small or very small (*m*≤0.12) migration rates in most cases but two (KO4, AVE; *m* = 24.9 and 3.07, respectively). Fu's *F*_S_ tests showed no significant deviations of *F*_S_ from values expected under neutrality (2.629>*F*_S_>−0.728; *P* always>0.206).

**Table 5:** Summary statistics of mismatch parameters of *C.kerkyrensis* mtDNA for demographic and spatial expansions.

		Demographic expansion parameters							Spatial expansion parameters						
	Obs. mean	*τ*	*T*	*θ_0_*	*N_0_*	*θ_1_*	*N_1_*	*P_SSD_*	*τ*	*T*	*θ*	*N*	*M*	*m*	*P_SSD_*
ALB	1.975	5.501	137,525	0.002	50	2.338	58,450	0.198	3.901	97,525	0.902	22,550	1.057	2.3×10^−5^	0.164
KO3	7.689	0.312	7,800	0.000	0	9999	2,5×10^5^	**0.001**	31.303	782,575	2.675	66,875	0.247	0.2×10^−5^	0.567
KO4	0.868	0.941	23,525	0.000	0	9999	2,5×10^5^	0.575	0.936	23,400	0.004	100	9999	24.9	0.428
AVE	0.668	3.000	75,000	0.000	0	0.510	12,750	0.457	0.334	8,350	0.065	1625	9999	3.07	0.117
CIR	5.256	1.871	46,775	0.002	50	5.328	133,200	0.070	1.819	45,475	0.001	25	6.263	0.12	0.061
FO	2.467	8.996	224,900	0.000	0	1.740	43,500	0.653	7.110	177,750	1.421	35,525	0.299	0.4×10^−5^	0.578
CP	1.000	3.000	75,000	0.000	0	0.133	3,325	**0.040**	5.628	140,700	0.001	25	0.281	0.006	0.262
Alb./S.Italy	3.926	7.695	192,375	0.002	50	6.450	161,250	0.091	6.006	150,150	0.906	22,650	0.906	0.2×10^−4^	0.488
Corfu Is.	3.714	0.576	14,400	0.627	15,675	9999	2,5×10^5^	0.483	0.575	14,375	0.627	15,675	0.627	0.2×10^−4^	0.385
C. Italy	7.256	9.226	230,650	0.002	50	11.057	276,425	0.098	5.656	141,400	2.602	65,050	2.602	0.2×10^−5^	0.243
All	25.656	46.258	11.5×105	0.000	0	66.663	16.6×10^5^	**0.005**	42.740	10.7×10^5^	5.579	139,475	5.279	1.9×10^−5^	0.107

Shown parameters are: the mismatch observed mean (Obs. mean), the expansion parameter (*τ*), time since the expansion (*T*), the mutation parameter (*θ*), the female effective population size (*N*), the scaled migration rate (*M*) and the immigration rate from neighboring demes (*m*). Estimates before (time 0) and after (time 1) expansion are given for the mutation parameter and for the female effective population size. *P_SSD_* (sum of squared deviations) is the probability of observing a less-good fit between the model and the observed distribution by chance. Bold *P* values are significant at the 0.05 level.

Table 6 and Figure 3 summarize the parameters of the isolation with migration model as obtained in the Bayesian coalescence-based method implemented in MDIV. Whatever generation time adopted (1 vs. 10 years), we always obtained large differences between TMRCA and *T* for all comparisons at any level; TMRCA is indeed from 1.7 (between Greek populations) to 5.6 (Central Italian vs. Greek populations) times higher than *T*. This evidence suggests isolation with low levels of gene flow (mean *M* = 2.9). The only exception to this otherwise generalized scenario is the comparison of Albanian+Southern Italian vs. Greek populations; here the difference between TMRCA and *T* is less extreme (TMRCA/*T* = 0.9) but *M* is still small (0.825), suggesting more recent divergence and no ongoing gene flow.

**Table 6:** Summary of parameters for the coalescence analyses in *C. kerkyrensis* at different geographical scales.

Comparisons	*N_ef_*	*T*	TMRCA
Within Albanian/Southern Italian haplogroup	8,705–87,050	22,450–224,501	10,085–100,850
Within Corfu Is.	15,322–153,220	38,382–383,820	22,238–222,380
Within Central Italian haplogroup			
CP-FO	9,987–99,870	25,017–250,170	10,000–100,000
CP-CIR	18,688–186,880	47,000–470,000	17,800–178,000
FO-CIR	15,418–154,180	38,915–389,150	21,795–217,950
Central Italy vs. Corfu Is.	28,575–285,750	73,152–731,520	13,025–130,250
Corfu Is. vs. Albania/Southern Italy	18,975–189,750	47,437–474,370	17,155–171,550
Albania/Southern Italy vs. Central Italy	21,237–212,370	53,199–531,990	15,870–158,700

*N_ef_* is the effective female population size; *T* is the time of population divergence and TMRCA is the expected time to the most recent common ancestor. Coalescence parameters were calculated using a generation time of 1–10 years and a mutation rate of 2% sequence divergence per million years (see Materials and Methods for details).

## Discussion

Here we present a comprehensive phylogeographic study on the fairy shrimp *C. kerkyrensis*, a species with a scattered Central - Mediterranean distribution and mostly bound to temporary ponds in low altitude oak or mixed woods, a habitat currently declining due to increasing urbanization and agricultural expansion [25]. Genetic data robustly show that the species is subdivided into three divergent mitochondrial lineages. Two of these lineages are very localized geographically (Central Italy and Corfu Is.) while a third one includes Southern Italian and Albanian populations. Hence, the Italian peninsula hosts two phylogeographically independent haplogroups.

It is important to stress that in the remainder of the study we will limit comparisons to studies centered on freshwater branchiopods only, because halophilic species often exhibit rates of molecular evolution faster than those of their freshwater counterparts [31]; this would severely hamper the reliability of any conclusion derived across these ecologically different categories.

### Molecular Systematics

Based on slight morphological differences in male antennae and in the ornamentation of female abdominal segments observed in samples collected in Central Italian ponds, these populations were assigned to the subspecies *C. kerkyrensis stellae* [32], which was subsequently elevated by the same author to the species rank (*C. stellae*) [32]. By increasing the number of study populations and individuals those characters were shown to display considerable intrapopulation variability and, hence, to have no taxonomic value [19]. In the same study *C. stellae* was synonymized with *C. kerkyrensis*; later on this systematic arrangement was further recognized as valid [4], [33].

We have molecularly identified three major allopatric and divergent lineages. Before processing our samples, we checked adult males and females for variation in the above mentioned morphological characters and we found them to be highly variable both within and between populations without the evidence of any clear pattern (either geographical or matching the genetic one) (Marrone, unpublished data). Sequence divergence among major haplogroups (uncorrected-p values) ranges from 1.1% (Albania+Southern Italy vs. Central Italy) to 3.2% (Central Italy vs. Greece) for the 16S gene and between 2.5% (Albania+Southern Italy vs. Greece) and 5.3% (Central Italy vs. Greece) for COI. These values encompass those obtained for 14 populations of the fairy shrimp *Tanymastix stagnalis* by using the same mtDNA markers (average values of 3.5% and 3.9% for 16S and COI, respectively) [27]. Sequence divergence among eight species of *Branchinella* ranges between 5% and 10% for 16S [34]. A COI-based phylogeny of the Italian species belonging to the genus *Chirocephalus* (including a single population of *C. kerkyrensis*) returned an average interspecific genetic distance value of 10.5% [26]. A comprehensive review of the COI barcoding data available for a variety of crustaceans revealed two non-overlapping ranges (0.46%–2.81% and 5.7%–25.3%) for intra- and interspecific comparisons [35]. Our figures for *C. kerkyrensis* are definitively close to the lower values. We conclude that the different lineages identified in this study constitute regional clusters within the species phylogeography; our data hence give further support to the current taxonomic arrangement [19]. Previous inconsistencies were likely due to the notorious difficulty in pinpointing taxonomically reliable morphological characters in anostracans. Indeed, these crustaceans seem prone to convergent evolution [28], [34], [36]–[40] also when characters alternative to the traditional ones, such as patterns of ornamentation of cyst shell, are taken into consideration [41]–[45].

### Phylogeography and historical demography

*C. kerkyrensis* has almost exclusively been recorded in temporary ponds in Mediterranean lowland oak or mixed woods, predominantly close to the coast. The Southern Italian location is the only exception to this otherwise generalized pattern in that is the single known pond where the species occurs at an altitude higher than 130 m above the sea level (ca. 500 m) [23]. Southern Italian and Albanian populations are connected in the haplotype network (Figure 2B) via the rare Southern Italian haplotype H6, which is just two mutational steps away from the most common Albanian haplotype (H2). Fairy shrimps rely on passive aerial movements of cysts to disperse among ponds [46]. Temporary pond communities are thought to be largely regulated according the principles of island biogeography, where elevation is often second only to distance in determining the overall species richness [47]. We hence hypothesize that an explanation for the occurrence of *C. kerkyrensis* at an altitude atypical for the species should be sought in occasional long-distance dispersal(s) of cysts carried inland by wind and/or migratory birds [23]; such a phenomenon has been already inferred molecularly in other diapausing aquatic invertebrates [48], [27], [28].

The overall pattern of genetic structuring in *C. kerkyrensis* can be fit into the Avise's phylogeographic category I [49], with spatially localized haplogroups separated by relatively profound genetic distances. The Southern Italian/Albanian haplogroup apparently deviates from this pattern. However, it can be reconciled with it if we consider AVE as an occasional derivate of ALB; the *F*_ST_ value between ALB and AVE (0.508) is indeed comparable to that among Central Italian populations (*F*_ST_ = 0.446±0.13) regardless of the very different geographical scale (Table 3).

Phylogeographic studies in European freshwater branchiopods have consistently retrieved patterns of extreme subdivision and large degrees of regionalism [26], [27], [28], [50], [51], [52]. This applies particularly well to the distribution area of *C. kerkyrensis* and, more generally, to the whole circum Mediterranean region, which allowed persistence in time of ancient (i.e. pre-glacial) lineages and, hence, pronounced genetic subdivision to develop [53]. To this historical perspective one should add the very limited potential of the species to sustain ongoing, recurrent gene exchanges among populations over long distances. Very limited amount of ongoing gene flow had been already detected among Central Italian populations of *C. kerkyrensis* by using 22 allozymic loci [26].

Our data could never reject a model of spatial expansion neither at the population nor at the haplogroup level, while demographic expansion was rejected for two populations (KO3 and CP). This suggests that mismatch distributions are supporting past fluctuations in range size better than past demographic fluctuations of populations. When demographic and spatial expansions are both supported, time estimates since last demographic expansion generally predate estimates since last spatial expansion (Table 5 and Figure 3). These time assessments must be considered cautiously though, because they are based on rates of substitutions, an issue that remains controversial in anostracans [31]. Nonetheless, the temporal shift between demographic and spatial expansions holds regardless of their absolute ages.

Demographic expansions are consistently placed during warm, interglacial phases of the Pleistocene that witnessed an expansion of oak forest in the Mediterranean region [54]. Spatial expansions were restricted to a temporal window narrower than that of the demographic ones and took place during cold periods (Figure 3), dominated by more open discontinuous vegetation [55]. One would be instinctively inclined to think that the species expanded its range when favorable habitats (Mediterranean plain forests) were flourishing. We rather believe that these conditions did not necessarily increase the chances for movements of individuals over medium and long distances and, thus, the establishing of new populations. Given the essentially random and opportunistic nature of dispersal in the group, which mostly relies on passive transport of cysts by birds, we argue that less forested surroundings would increase the probability of ponds to be spotted and, hence, visited by waterbirds. It has been clearly demonstrated that waterbirds are more attracted by ponds situated in open habitats than by those surrounded by forests or shrubs [56], [57]. Could it possibly be due to just a mere chance that the single study documenting a weak phylogeographic structure in a fairy shrimp species focuses on an area dominated by open grasslands [58]?

Demographic expansion started always with severe reduction in population size. Spatial expansion, regardless whether considering single populations or groups of populations, was always sustained by small or very small rates of exchange with neighboring populations (see *m* values in Table 5). The small effective female population sizes from which demographic expansions started, together with the low rates of gene flow required to explain range expansions, suggest a scenario where colonization of new areas proceeded essentially through the establishment of new populations via occasional movements of migrants (cysts) from nearby areas. The MDIV analyses for the two hierarchical levels considered in the study (within and between haplogroups) consistently found large differences between time of population divergence (*T*) and time to the most common ancestor (TMRCA), with the former being always higher than the latter, a pattern that suggests past isolation with low levels of migration *M* (Table 6 and Figure 3). When the geographical scale is narrowed down to nearby locations, the spatial autocorrelation analysis suggests that non-random positive genetic structure can only be observed if the distance among populations is less than 23 km; no IBD pattern is detectable in the data set. Indeed, only the two geographically very close Greek populations share haplotypes.

All this evidence taken together would explain the high divergence among populations observed in this study, because I) the effect of genetic drift is larger in small populations and II) time to fixation of local genetic variants is inversely proportional to effective population size.

The emerging scenario is thus one where dispersal of cysts works successfully across small distances only (with the sole exception of the Southern Italian/Albanian haplogroup). In light of the above considerations, of the fact that the range of the species is naturally fragmented and that a number of ponds where the species was known to occur in the past have recently vanished [25] (Figure 1), we conclude that *C. kerkyrensis* seems prone to experience depletion of its original gene pool. The presence of phylogeographic gaps not only among the major haplogroups but also at a very regional scale (Central Italy and Corfu Is.) suggests that this phenomenon might already have begun. As a matter of fact, extinctions of populations harboring intermediate haplotypes may result in the appearance of phylogeographic gaps within as well as between populations [49]. This phenomenon could be particularly exacerbated in *C. kerkyrensis* owing to its ability to maintain gene flow over short distances only (less than 23 km as suggested by the spatial autocorrelation analysis for Central Italy).

On the other hand, we cannot exclude that further samplings in the Balkan area whose temporary water bodies are largely unexplored, would return additional populations of the species, as recently happened with the Albanian site, and, hence, would possibly lead to partially reconsider the phylogeographic scenario proposed here. More generally, the questions left open in this study are the result of a lack of knowledge on the distribution of Southern European anostracans, which is still largely fragmentary and mostly reflects ranges of activity of the few experts of the group [1], [59], [60]. Detailed faunistic surveys are hence urgently needed, also in light of the threats Southern European temporary water bodies are faced with. All this being considered, the high level of mtDNA regionalism we have uncovered here strongly suggests that whatever efforts would be eventually undertaken to protect this declining species (or, better, its fragile habitat) should be determined at regional scales by carefully evaluating local ecological and evolutionary dynamics to avoid any further loss of diversity [58].

## Materials and Methods

### Sampling, DNA extraction, PCR amplification and sequencing

All known extant populations of *C. kerkyrensis* have been sampled for the study. Details on sampling localities and sample sizes are given in Table 1 and Figure 1. Total genomic DNA was extracted following [61], [62]. We used the same primer pairs, PCR cycling conditions and purification of PCR products as in [61], [62] to amplify about 700 and 500 base pairs (bp) of mitochondrial DNA (mtDNA) of the Cytochrome Oxidase I (COI) and 16s rRNA (16S) genes for each individual included in the study. PCR products were sequenced with an automated sequencer (Applied Biosystems 3130xl Genetic Analyzer) according to the manufacturer's protocols. To promote accuracy, strands were sequenced in either direction using the primer pairs employed for PCRs. The sequences have been deposited in GenBank (Accession No. JN246475-JN246530).

### Phylogenetic and Population genetics analyses

Sequences were edited and aligned using Sequencher 4.6 (Gene Code Corporation); alignments were also checked by eye. Phylogenetic analyses (see details below) were initially carried out on each gene region separately. Resulting topologies were almost identical in all cases (data not shown). The suitability of pooling sequence data from the two sequenced genes was further assessed by the incongruence length difference test (1,000 replicates) [63] as implemented in PAUP* 4.0b10 [64] with 100 random stepwise addition and TBR branch-swapping algorithm. The test showed that the two regions are not phylogenetically incongruent (*P* = 0.987); we therefore only report analyses based on the two genes combined. We used PAUP* 4.0b10 to calculate base frequencies and to test for base frequency homogeneity across taxa (*χ* ^2^ test).

We analyzed data phylogenetically with *Chirocephalus marchesonii* (Pilato Lake, Marche, Central Italy; 3 individuals) and *Eubranchipus* sp. (GenBank Accession Nos. AF209061.1 and AF209052.1 for COI and 16S, respectively) as outgroups. *C. marchesonii* is basal to *C. kerkyrensis* in a COI-based phylogeny of the genus [26]; *Eubranchipus sp.* was chosen to represent an additional genus within the family Chirocephalidae. We used MODELTEST [65] to determine the model of sequence evolution that best fit our data. We then used the MODELTEST output for the Maximum Likelihood (ML) analyses and to calculate ML genetic distances for the Neighbor-Joining (NJ) analyses; ML and NJ were carried out in PAUP. We employed the same model of sequence evolution for Bayesian searches. These were run in MrBAYES 3.0b4 [66] allowing site-specific rate variation partitioned by gene and, for COI, by codon position. MrBAYES was run for 2,000,000 generations with a sampling frequency of 100 generations. We ran one cold and three heated Markov chains and two independent runs. To establish if the Markov chains had reached stationarity, we plotted the likelihood scores of the sampled trees against generation time. Trees generated before stationarity were discarded as burn-in (first 10% of the sampled trees) and posterior probability values for each node were calculated on the basis of the remaining 90% of sampled trees. These trees were then used to construct a 50% majority rule consensus tree in PAUP. The robustness of the ML and NJ hypotheses was tested by 1,000 bootstrap replicates in PAUP.

We used the software TCS [67], which implements the statistical parsimony procedure [68] to derive a gene genealogy of the species. The program collapses sequences into haplotypes and produces a network linking haplotypes only if they have a 95% (or higher) probability of being justified by a parsimony criterion.

We employed ARLEQUIN 3.0 [69] to calculate the following parameters of genetic diversity for each population: haplotype diversity (*h*), mean number of pairwise differences between all pairs of haplotypes (*π*) and the nucleotide diversity (*π_n_*). Levels of genetic structure were initially tested with any *a priori* grouping of populations by a hierarchical analysis of molecular variance (AMOVA) in ARLEQUIN with 10,000 replications [70]. In case that significant genetic structure was found, we would then run a grouped AMOVA with the same number of replications as in the ungrouped one. We also used ARLEQUIN to calculate pairwise *F*_ST_ values for all pairs of populations; statistical significance of *F*_ST_ values was assessed by 10,000 permutations with Bonferroni correction for multiple tests.

To test for genetic similarity among individuals whose geographic separation falls within a given distance class we applied the spatial autocorrelation analysis. To take into consideration the interplay between distant class sizes and the true (but unknown) extent of spatial genetic structure the analysis was conducted on the following groups of populations at increasing distance class sizes: Central Italy (distance class size = 10 km), Central Italy+Corfu Is. (distance class size = 50 km), Albania+Southern Italy and all populations together (in both cases with a distance class size of 100 km). Each analysis was run in GENALEX 6 with 999 permutations; confidence intervals of the correlation coefficient (*r*) were estimated with 1,000 bootstrap replicates [71]. Finally, Isolation-by-distance (IBD) was tested by using the Mantel test as implemented in ARLEQUIN; the program correlates matrices of pairwise *F*_ST_ values and geographic distances among all pairs of populations.

ARLEQUIN was also used to assess the fit of demographic [72] and geographic [73] expansion models to fairy shrimp genetic variations (each population singularly; groups of populations as in the AMOVA design and all populations pooled together). Mismatch distributions were used to fit the model of sudden demographic expansion and to estimate its parameters. These included *τ* and the *θ*-estimates *θ_0_* = 2*N_0_μ* and *θ_1_* = 2*N_1_μ*, where *μ* is the branchiopod mtDNA mutation rate of 2% sequence divergence per million years [21] and *N_0_* and *N_1_* are the female effective population sizes before (time 0) and after (time 1) expansion. Mismatch distributions were also employed to fit the infinite island model [74] and to estimate the relative parameters, which included *τ*, *θ* = *θ_0_* = *θ_1_* and *M* = 2*Nm* (*θ* is 2*Nμ* at demographic equilibrium and *m* is the rate at which the sampled deme would exchange migrants with a unique population of infinite size after *T* generations). In both models *τ* is a mutation-scaled measure of time since expansion (demographic or geographic) and can be used to estimate time in generations (*T*) since expansion, using *T* = *τ*/2*μ* (where *μ* is the mutation rate). Goodness of fit was assessed by the sum of square deviations (SSD) between the observed and the expected mismatch, and its significance was determined by a parametric bootstrap with 10,000 replicates. In addition, we used the Fu's *F*_S_ test [75] to test the neutrality of our data set. We compared *F*_S_ values against a distribution generated from 10,000 random samples under the null hypothesis of selective neutrality. Negative and significant *F*_S_ values are taken as an evidence of deviation from neutrality.

Bayesian sampling coalescence-based methods implemented in MDIV [76] were used to estimate the “isolation with migration” parameters *θ* = 2*N_ef_μ* where *N_ef_* is the effective female population size and *μ* is the mutation rate, migration rate (*M* = 2*N_ef_*m), time of population divergence from pairwise-sample comparisons (*T* = *t*/*N_ef_*), and the expected time to the most common recent ancestor (TMRCA = *tμ*). Assuming isolation to prevail in most cases over migration, we did not attempt to estimate directionality of gene flow. The model allows distinguishing between a past isolation with recent migration (probably after secondary contact) and a recent isolation with low or any migration [76]. In the first scenario, the TMRCA will be high whereas the divergence will be low and differences between *T* and TMRCA are small. In the second scenario, there will be broader differences between *T* and TMRCA with *T* being consistently higher than TMRCA and levels of migration will be low. The program was run under a finite sites model (HKY), using five independent Markov chains with 5,000,000 steps and an initial 10% burn-in. The best estimates of *θ*, *M* and *T* were those displaying the highest posterior probabilities. Comparisons were carried out at two hierarchical levels: (1) between each possible pair of groups identified phylogenetically and phylogeographically (same groups as in the AMOVA) and (2) between geographically adjacent populations. Here, all possible pairwise comparisons among populations within the following areas were considered: Central Italy, Corfu Is., and Southern Italy+Albania. Values for *N_ef_*, *t*, and TMRCA were calculated using a generation time of 1–10 years and a mutation rate as above. The choice of such broad generation time range derives from the fact that no precise data on generation time in *C. kerkyrensis* are available. Moreover, the one- year generation time usually adopted in temperate systems is questionable for organisms capable to produce resting eggs, which can store viable propagules for many years [77].
